# Seamless growth of a supramolecular carpet

**DOI:** 10.1038/ncomms10653

**Published:** 2016-02-03

**Authors:** Ju-Hyung Kim, Jean-Charles Ribierre, Yu Seok Yang, Chihaya Adachi, Maki Kawai, Jaehoon Jung, Takanori Fukushima, Yousoo Kim

**Affiliations:** 1 Surface and Interface Science Laboratory, RIKEN, 2-1 Hirosawa, Wako, Saitama 351-0198, Japan; 2 Department of Chemical Engineering, Pukyong National University, 365 Sinseon-ro, Nam-gu, Busan 608-739, Republic of Korea; 3 Center for Organic Photonics and Electronics Research (OPERA), Kyushu University, 744 Motooka, Nishi, Fukuoka 819-0395, Japan; 4 Department of Advanced Materials Science, The University of Tokyo, 5-1-5 Kashiwanoha, Kashiwa, Chiba 277-8561, Japan; 5 Department of Chemistry, University of Ulsan, 93 Daehak-ro, Nam-gu, Ulsan 44610, Republic of Korea; 6 Chemical Resources Laboratory, Tokyo Institute of Technology, 4259 Nagatsuta, Midori-ku, Yokohama 226-8503, Japan

## Abstract

Organic/metal interfaces play crucial roles in the formation of intermolecular networks on metal surfaces and the performance of organic devices. Although their purity and uniformity have profound effects on the operation of organic devices, the formation of organic thin films with high interfacial uniformity on metal surfaces has suffered from the intrinsic limitation of molecular ordering imposed by irregular surface structures. Here we demonstrate a supramolecular carpet with widely uniform interfacial structure and high adaptability on a metal surface via a one-step process. The high uniformity is achieved with well-balanced interfacial interactions and site-specific molecular rearrangements, even on a pre-annealed amorphous gold surface. Co-existing electronic structures show selective availability corresponding to the energy region and the local position of the system. These findings provide not only a deeper insight into organic thin films with high structural integrity, but also a new way to tailor interfacial geometric and electronic structures.

## Abstract (web summary)

The potential of organic molecular devices are limited by our inability to uniformly arrange them as thin films on metals, especially over imperfect surfaces. Here, the authors demonstrate the growth of a self-organized molecular carpet, which can extend over steps and terraces while retaining regularity.

## Graphical abstract

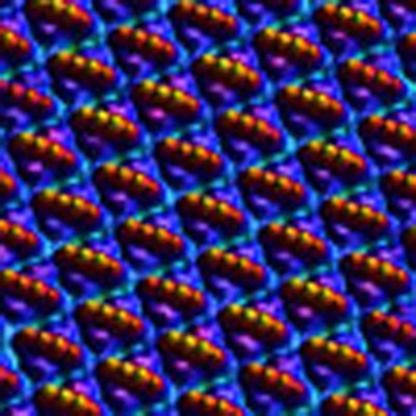

Organic thin films (OTFs) have received much attention for their potential in various electronic and optoelectronic device applications, since they have outstanding advantages (that is, low cost, low weight and mechanical flexibility) in comparison with standard inorganic technologies^1^,^2^,^3^,^4^,^5^,^6^. In particular, it has been recognized in recent years that organic/metal (O/M) interfaces at which charge carriers are injected into OTFs play a crucial role in the operation and performance of organic devices^7^,^8^,^9^,^10^,^11^,^12^,^13^,^14^. Various interactions at the O/M interface, such as surface–molecule and intermolecular interactions, are of great importance in the formation of intermolecular networks and organic epitaxy, and have a strong correlation with their electronic structures^7^,^9^,^13^,^15^,^16^,^17^,^18^,^19^. Although the purity and uniformity of OTFs at the O/M interfaces have profound effects on the operating characteristics of organic devices^7^, the formation of OTFs with high interfacial uniformity on metal surfaces has suffered from the intrinsic limitation of molecular ordering imposed by surface step edges, due to the relatively weak intermolecular interactions between organic molecules. Extensive research efforts in the development of OTFs with high interfacial uniformity on metal surfaces have mostly focused on examining the formation of covalent bonding and supramolecular complexes between organic precursors via laborious post-treatments, such as epitaxial surface polymerization and metal–organic coordination^20^,^21^,^22^,^23^,^24^,^25^. Recently, one-dimensional (1D) molecular assembly across step edges was achieved on a specific metal surface via hydrogen bonding^26^,^27^, and at the solid–liquid interface, the formation of supramolecular networks with high uniformity on rough graphene has been studied in depth^28^. However, the creation of widely uniform OTFs on metal surfaces that maintain structural integrity based on non-bonding intermolecular interactions remains a challenge for further development and enhancement of organic devices fabricated by one-step deposition of organic molecules.

In this article, we successfully demonstrate the formation of a supramolecular carpet (SMC), with a widely uniform interfacial structure and high adaptability, on a metal surface, via a one-step deposition process. The geometric and electronic structures of the SMC were investigated by means of scanning tunnelling microscopy/spectroscopy (STM/STS) and density functional theory (DFT) calculations. For this work, bis[1,2,5]thiadiazolotetracyanoquinodimethane (BTDA-TCNQ)^29^ was used as the key building block with which to realize the SMCs, not only on a single crystal gold (Au) surface but also on a pre-annealed amorphous Au surface. Tetracyanoquinodimethane (TCNQ) is one of the strongest organic electron acceptors to have found wide use in organic devices, and forms a strongly bonded donor–acceptor complex with tetrathiafulvalene (TTF)^17^,^19^,^30^,^31^,^32^. Thus, we used a model system of BTDA-TCNQ, which integrates the structural properties of both TCNQ and TTF in a single molecule, for realizing self-organization into an ordered domain. BTDA-TCNQ is a TCNQ derivative fused with 1,2,5-thiadiazole rings, exerting strong intermolecular interactions in a planar fashion to form network structures in the single crystal^29^ and even in its charge-transfer crystals^33^,^34^. The rhombic structure of BTDA-TCNQ exhibits twofold symmetry with two mirror planes, allowing perpendicular alignment of two electrostatically opposite symmetry axes consisting of electronegative and electropositive end groups, respectively. It, therefore, enables equivalent intermolecular interactions along the four sides of the molecule. Such rhombic structure also facilitates access of neighbouring molecules to each other via high electrostatic interactions in the four directions, which leads to topographically favourable intermolecular interactions. The strong non-bonding intermolecular interactions, balanced with surface–molecule interfacial interactions and site-specific rearrangements of the BTDA-TCNQ molecules near surface step edges, enable the step-flow growth mode for the SMC formation^35^,^36^. This can lead to an extension of the SMC over step edges of the Au surface without loss of its structural integrity, and results in a covering with high interfacial uniformity over multiple surface steps and terraces, even on the pre-annealed amorphous Au surface prepared on a glass substrate. In addition, different types and dimensionalities of the interfacial electronic structures are distinctively observed in the SMC of BTDA-TCNQ on the Au(111) surface, corresponding to the energy region and the local position of the system, implying that various types of electronic structures projected onto the SMC can be selectively accessible. These results suggest that the SMC has great potential for applications in organic electronics, and also provide important guidelines to develop novel materials forming seamless OTFs on various surfaces.

## Results

### Intermolecular network of BTDA-TCNQ/Au(111)

**Figure 1:**
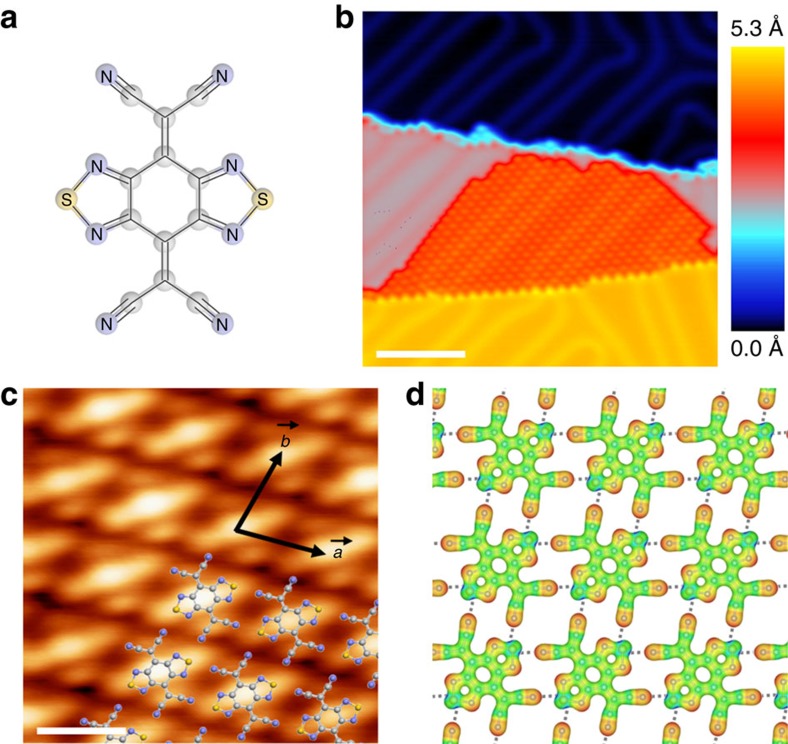
Network structure of the BTDA-TCNQ molecules. (**a**) The chemical structure of BTDA-TCNQ. (**b**) STM image of BTDA-TCNQ/Au(111), which shows that the molecular network of BTDA-TCNQ grows from the step edge at the lower terrace (sample bias voltage (*V*_s_)=1,000 mV, tunnelling current (*I*_t_)=1.00 nA, scale bar (*S*), 10.0 nm). The reconstructed herringbone structures of Au(111) beneath the BTDA-TCNQ molecules are clearly evident. Height scale is indicated in colour, and the step height of Au(111) is found to be around 2.3 Å. (**c**) Close-up STM image of the network structure formed by BTDA-TCNQ (*V*_s_=2,000 mV, *I*_t_=1.0 nA, *S*=1.0 nm). Molecular models reveal that the unit cell is noticeably similar to that of the coplanar molecular network in the crystal structure of BTDA-TCNQ. (**d**) The electrostatic potential map of the BTDA-TCNQ network. Blue to red corresponds to positive to negative charges. The distance for the S–N contacts (indicated by grey dotted lines) is 2.80 Å, and the calculated binding energy is ∼0.70 eV per molecule.

On deposition of BTDA-TCNQ on the Au(111) surface, decoration at the step edge occurs first, and then growth of a highly ordered SMC proceeds from the step edge across the lower terrace without further nucleation occurring on the terrace (see Fig. 1a,b). This step-flow growth mode of the SMC exhibits a quasi-epitaxial nature with an in-plane azimuthal orientation of BTDA-TCNQ (see Supplementary Figs 1 and 2). The reconstructed herringbone structures of Au(111) beneath the SMC are readily observed in the STM images, which reveals that the adsorption of BTDA-TCNQ on the Au(111) terraces does not involve quenching of the Au(111) Shockley surface state, as will be discussed below in relation to the STS spectra. These experimental observations indicate that the surface–molecule interfacial interactions of BTDA-TCNQ/Au(111) are sufficiently weak so that lateral diffusion of the molecules across the Au(111) terraces is possible^35^,^36^. Contrary to the weak adsorption characteristics of BTDA-TCNQ/Au(111), the partial positive and negative charges of the S and N atoms of BTDA-TCNQ, respectively, introduce effective attractive interactions (that is, the S–N intermolecular interactions) between the neighbouring molecules, which determine the array of the molecules in the SMC, as shown in Fig. 1c,d. Detailed analysis of the STM images (see Fig. 1c) gave lattice constants for the SMC unit cell of *a*=*b*=10.0±1.0 Å and *γ*=80±5°, which are noticeably similar to those achieved by the S–N intermolecular interactions of the coplanar molecular network in the single crystal structure of BTDA-TCNQ^33^,^34^.

DFT calculations were also performed to examine the formation of SMC and to confirm its stability in terms of intermolecular interactions. On the basis of the experimental observations of weak surface–molecule interactions, the substrate was not considered in the calculations. For comparisons among a variety of initial geometries, various molecular orientations rotated by 10° increments were considered with the experimentally observed periodicity of the intermolecular network structure. On the basis of our computational approach, three non-equivalent local potential minima were found corresponding to the varied molecular orientations (see Supplementary Fig. 3). The experimentally observed azimuthal orientation is most favourable with the highest binding energy per molecule of ∼0.70 eV (see Fig. 1d), which is more stable than the other two local potential minima by >0.30 eV. Note that the calculated binding energy of ∼0.70 eV per molecule is highly competitive with the dipole polarization energy arising from donor–acceptor complexation in the charge-transfer crystal^37^,^38^. The higher stability of the geometric structure of the SMC, in comparison with the followed second and third local potential minima, mainly originates from the relative orientations of electronegative cyano (–CN) groups between the neighbouring molecules. The electrostatic repulsions between the –CN groups are easily expected in both second and third local potential minima rather than the S–N intermolecular interactions (see Supplementary Fig. 3), which implies that the S–N intermolecular interactions are an impetus for the formation of SMC. The calculated lattice constants of the SMC unit cell (*a*=*b*=9.9 Å and *γ*=81.5°) are almost identical to our STM observations, and the calculated distance for the S–N contacts (∼2.80 Å) is also well matched with that of the crystal structure of BTDA-TCNQ (∼3.04 Å; ref. 29).

### Seamless growth of SMC with high structural integrity

**Figure 2:**
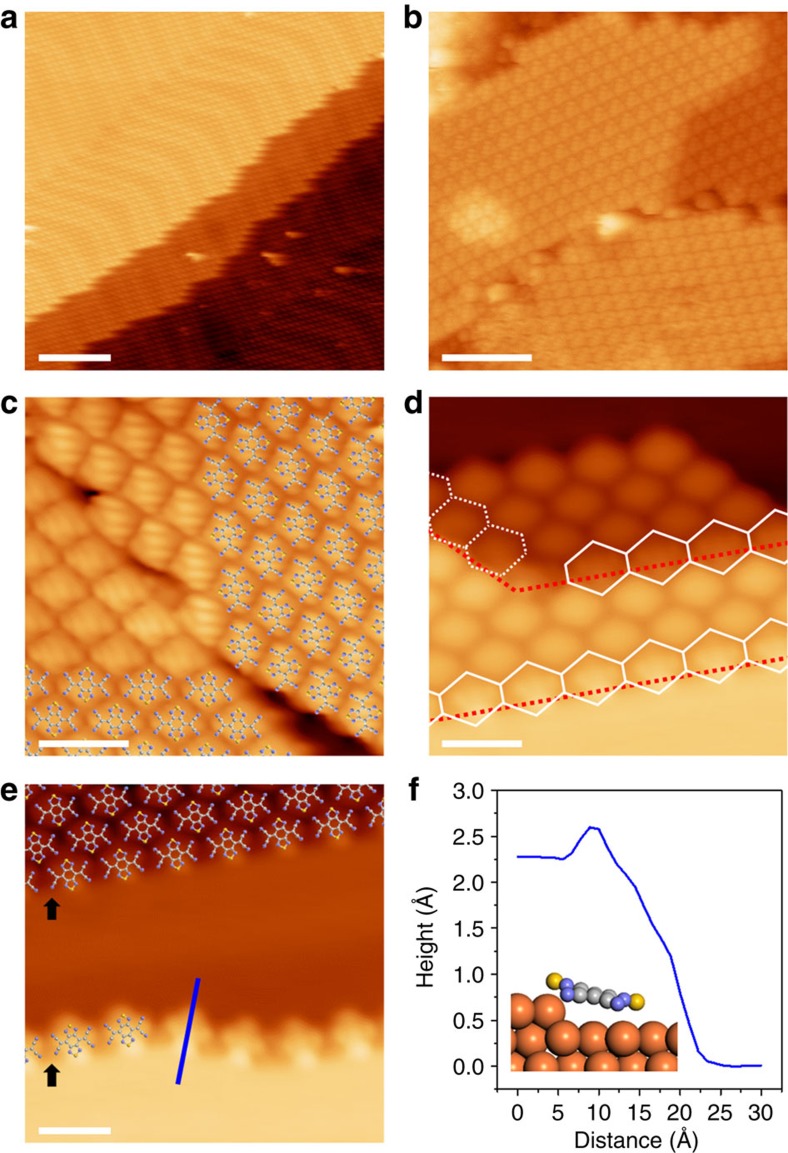
Highly ordered SMC of BTDA-TCNQ on the Au surfaces. (**a**) STM image of the high-coverage regime of BTDA-TCNQ on Au(111) (*V*_s_=2,000 mV, *I*_t_=0.5 nA, *S*=12.0 nm), showing that the highly ordered SMC of BTDA-TCNQ is extended over multiple surface steps and terraces without losing its structural integrity. (**b**) STM image of an SMC of BTDA-TCNQ on the pre-annealed amorphous Au surface (*V*_s_=2,000 mV, *I*_t_=0.3 nA, *S*=5.0 nm). (**c**) STM image of a structural defect in an SMC of BTDA-TCNQ between two misaligned domains on Au(111) (*V*_s_=1,000 mV, *I*_t_=0.1 nA, *S*=2.0 nm). (**d**) STM image of an SMC of BTDA-TCNQ of which the structural integrity is not interrupted by the surface step edge of Au(111) (*V*_s_=300 mV, *I*_t_=0.5 nA, *S*=2.0 nm). The BTDA-TCNQ molecules with the slanting adsorption structure (enclosed with white solid lines) and the rearranged flat-lying adsorption structure (enclosed with white dotted lines) are clearly shown near the step edges of Au(111) (indicated by red dotted lines). (**e**) STM image of the BTDA-TCNQ molecules which lean down from the upper terrace to the lower terrace at the step edge (*V*_s_=500 mV, *I*_t_=0.5 nA, *S*=2.0 nm). The step edges of Au(111) are indicated by black arrows. (**f**) The height profile along the blue line in **e**. corresponding to the slanting adsorption structure of BTDA-TCNQ at the step edge is presented here. The slanting adsorption structure of BTDA-TCNQ is schematically illustrated in the inset of **f**.

In the high-coverage regime of BTDA-TCNQ shown in Fig. 2a, the SMC grows very compactly, covering the whole Au(111) surface, and is extended over multiple surface steps and terraces in a downhill direction, without losing its structural integrity. It is worth noting that single molecular domains of the SMC with a few hundred nanometres in length and width could be achieved on the Au(111) surface, of which sizes are meaningfully large when recent lithography techniques are considered for further applications^39^,^40^. Interestingly, the outstanding growth characteristics of BTDA-TCNQ are maintained on the pre-annealed amorphous Au surface, as shown in Fig. 2b. The amorphous Au surface was prepared on the cleaned glass substrate by thermal evaporation in a vacuum chamber, and followed by a thermal annealing process without any other preliminary treatments. Note that the amorphous Au surface tends toward the close-packed (111) facet with irregular steps and terraces after annealing, because Au(111) is the most thermodynamically stable facet of Au (see Supplementary Fig. 4). Although structural defects of the SMC, such as domain boundaries and dislocations, are occasionally observed between misaligned domains on Au(111), the structural integrity is not essentially interrupted by the step edges (see Fig. 2c,d). These results imply that the S–N intermolecular interactions in the molecular ordering of BTDA-TCNQ remain intact across the surface steps. This feature distinguishes the behaviour of the BTDA-TCNQ system from that of most other supramolecular assemblies on metal surfaces involving the structural complexities near surface step edges. The adsorption structure of BTDA-TCNQ at the step edges plays a decisive role in maintaining the S–N intermolecular interactions between the two adjacent terraces, thus preserving the structural integrity of the SMC over multiple surface steps and terraces. When the BTDA-TCNQ molecules initially decorate the step edges, they lean down from the upper terrace to the lower terrace (that is, the slanting adsorption (SA) structure), as shown in Fig. 2e,f. The STM observations indicate that the S–S molecular axis (the molecular axis along the two S atoms) of BTDA-TCNQ in this structure is perpendicular to the step line, and thus the two S atoms are located on the upper and lower terraces, respectively (see Fig. 2e,f). In consideration of the symmetry of BTDA-TCNQ, the SA structure enables symmetrical intermolecular interactions on the upper and lower terraces, leading to the intact intermolecular interactions to be valid across the surface steps. Differently from the weak adsorption nature of BTDA-TCNQ on the Au(111) terraces, the surface–molecule interfacial interactions localized near the step edges are the primary factors that determine the SA structure, as will be discussed in relation to the DFT calculations and the STS spectra.

For the BTDA-TCNQ molecules that are aligned along parallel straight lines of the step edges, the SA structure at the step edges is preserved even in the high-coverage regime (see Fig. 2d), thus acting as a bridge for maintaining the S–N intermolecular interactions across the adjacent terraces. However, the BTDA-TCNQ molecules that are adsorbed out of the parallel straight lines at the step edges are displaced from step edges and rearranged onto the lower terraces (that is, the rearranged flat-lying adsorption structure), as shown in Fig. 2d. These phenomena imply that the S–N intermolecular interactions in supramolecular assembly dominate the surface–molecule interactions even at the relatively reactive step edges, and thus result in the SMC formation with structural integrity maintained over wide areas. In addition, the potential energy barrier for the diffusion of molecule crossing the upper step edge is significantly higher than the lower step edge, because the step dipole is mostly formed below the upper step edge^41^,^42^,^43^. The SMC, therefore, grows across the surface steps only in the downhill direction.

### Adsorption structure of BTDA-TCNQ at the step edges

**Figure 3:**
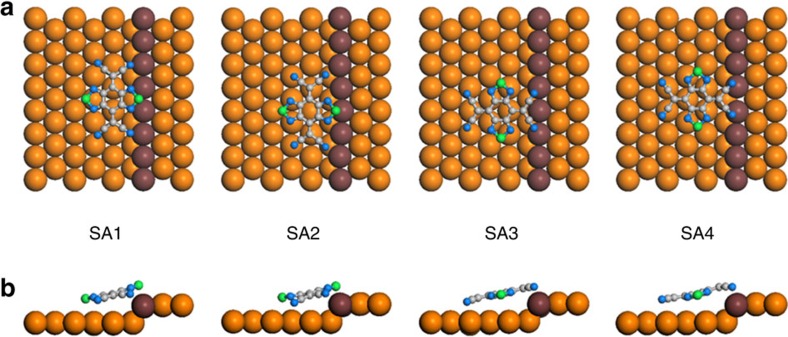
DFT calculations for the SA structure of BTDA-TCNQ at the step edge of Au(111). (**a**,**b**) Optimized geometries of four different hypothetical model structures of BTDA-TCNQ adsorbed at the step edge of Au(111) (designated ‘SA1', ‘SA2', ‘SA3' and ‘SA4', respectively) by DFT calculations. The S–S molecular axes of the molecules are perpendicular to the step line in ‘SA1' and ‘SA2', and parallel in ‘SA3' and ‘SA4'. Side views of **a** are shown in **b**.

To gain deeper insight into the SA structure of BTDA-TCNQ at the step edges, DFT calculations were performed in consideration of four different adsorption models (designated ‘SA1', ‘SA2', ‘SA3' and ‘SA4', respectively) as shown in Fig. 3 and Table 1. Among these structure models, the most stable adsorption structure of the BTDA-TCNQ molecule at the step edge was found to be ‘SA1' with the adsorption energy of 2.64 eV. In ‘SA1', the S–S molecular axis of BTDA-TCNQ is perpendicular to the step line, and the two S atoms are located on the upper and lower terraces, respectively. Compared with ‘SA3' that is the most stable adsorption structure with the S–S molecular axis parallel to the step line, ‘SA1' shows higher adsorption energy by 0.52 eV, which is consistent with the experimental observations of the SA structure at the step edges.

The detailed geometric interpretation of the computational results indicates that the –CN groups of the BTDA-TCNQ molecule significantly contribute to determination of the adsorption structure at the step edges. Side views of the molecular adsorption structures clearly reveal that the –CN groups are displaced from the molecular plane and become closer to the surface, compared with the S atoms (see Fig. 3b). In the most stable ‘SA1', the nearest atomic distance between the molecule and the reactive Au atoms of the step edge (–CṄ̇̇Au_edge_) is 2.18 Å, and is much shorter than the Ṡ̇̇Au_edge_ distance of 3.12 Å even though the S atom of BTDA-TCNQ is interacting with the two Au_edge_ atoms. The higher stability of ‘SA1' than ‘SA3' also can be deduced from the shorter –CṄ̇̇Au_edge_ distance in ‘SA1' than that of ‘SA3' by 0.13 Å. Combined with the –CṄ̇̇Au_edge_ interactions, the relatively weak Ṡ̇̇Au_edge_ interactions in ‘SA1' also possibly participate in determining the adsorption structure when no existing Ṡ̇̇Au_edge_ interaction in ‘SA3' is considered. In addition, the charge density difference map of the most stable ‘SA1' structure clearly reveals the partial charge transfer from the surface to the molecule through the –CN groups at the step edge (see Supplementary Fig. 5), and the corresponding amount of the partial charge transfer evaluated by Bader population analysis is 0.68*e*. The computationally estimated feature of the partial charger transfer strongly supports the surface–molecule interfacial interactions localized at the step edges, resulting from the step dipole as will be discussed in relation to the STS spectra.

### Electronic structures of BTDA-TCNQ on the Au surface

**Figure 4:**
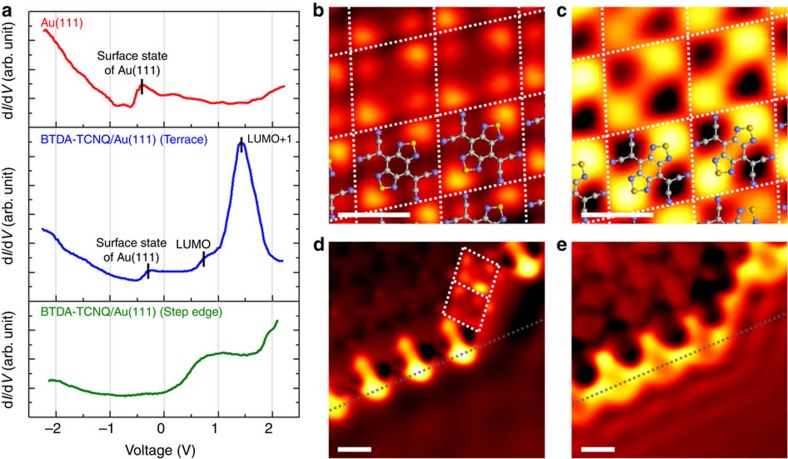
STS spectra and STS mapping images of BTDA-TCNQ/Au(111). (**a**) STS spectra of (top) the bare Au(111) surface, (middle) the BTDA-TCNQ molecule on the Au(111) terrace and (bottom) the BTDA-TCNQ molecule at the Au(111) step edge. (**b**,**c**) STS mapping images of BTDA-TCNQ on the Au(111) terrace. The electron probability distributions are clearly observed for the LUMO state of BTDA-TCNQ in **b** (*V*_s_=800 mV, *I*_t_=0.8 nA, *S*=1.0 nm) and the LUMO+1 state of BTDA-TCNQ in **c** (*V*_s_=1,500 mV, *I*_t_=0.8 nA, *S*=1.0 nm). Each BTDA-TCNQ molecule is enclosed with white dotted lines in **b** and **c**. (**d**,**e**) STS mapping images of BTDA-TCNQ at the Au(111) step edge. Parallel straight lines of the step edge are indicated by grey dotted lines in **d** and **e**, and the BTDA-TCNQ molecules that are rearranged onto the lower terrace from the step edge are enclosed with white dotted lines in **d**. The STS mapping image in **d** (*V*_s_=500 mV, *I*_t_=1.0 nA, *S*=1.0 nm) reveals the LUMO state for BTDA-TCNQ with the rearranged flat-lying adsorption structure and the LUMO+1 state for BTDA-TCNQ with the slanting adsorption structure at the lower sample bias than that in **b** and **c**. The STS mapping image in **e** (*V*_s_=2,000 mV, *I*_t_=1.0 nA, *S*=1.0 nm) also indicates that the unoccupied MO states of BTDA-TCNQ are highly hybridized with the Au surface at the step edge, forming quasi-1D electron dispersion along the step-edge line.

To elucidate the electronic structures of BTDA-TCNQ on the Au surface in more detail, intensive STS and STS mapping measurements were performed, as shown in Fig. 4. The Au(111) Shockley surface state, the band edge of which is ∼500 meV below the Fermi level (*E*_F_), remains clearly evident in the STS spectrum of the BTDA-TCNQ molecule adsorbed on the Au(111) terrace, indicating the weak adsorption character of BTDA-TCNQ/Au(111) (refs 44, 45). The lowest unoccupied molecular orbital (LUMO) and the LUMO+1 states of BTDA-TCNQ are distinctly observed in the STS spectrum (at ∼+800 mV and ∼+1,500 mV, respectively). STS mapping reveals the electron probability distributions for these unoccupied molecular orbital (MO) states (see Fig. 4b,c), demonstrating that the LUMO state is locally distributed around the –CN groups and the S atoms, forming a rhombic structure, whereas the LUMO+1 state shows a more conjugated electronic structure along the S–S molecular axis. In general, the unoccupied MO states of BTDA-TCNQ are largely distributed around the S atoms, even though their electronic structures are distinct from each other. The conjugated character of the unoccupied MO states of BTDA-TCNQ results in n-type semiconducting behaviour, which is clarified in a field-effect transistor (FET) configuration (see Supplementary Fig. 6). Note that the distinct electronic structures of the unoccupied MO states lead to strong bias dependence in STM imaging in positive sample bias region (see Supplementary Fig. 1), because STM imaging strongly reflects the integration of the electronic states that can contribute to electron tunnelling^46^.

At the step edge of Au(111), however, the STS spectrum indicates a rather strong interaction between BTDA-TCNQ and the Au surface. The unoccupied feature of BTDA-TCNQ at the step edge shows a steep initial rise near the *E*_F_, and then maintains a nearly constant level before another steep rise at ∼+1,700 mV. Such substantial broadening of the unoccupied states is mainly caused by hybridization with the electronic states of the metal surface, and thus the feature for the step-edge state of Au(111) is not distinguishable in the STS spectrum of BTDA-TCNQ adsorbed at the step edge^42^. STS mapping reveals that the unoccupied MO states of the BTDA-TCNQ molecules show a significant downward shift toward the *E*_F_ at the step edge. The LUMO+1 state for the SA structure and the LUMO state for the rearranged flat-lying adsorption structure of BTDA-TCNQ at the step edge are observed at ∼+500 mV, as shown in Fig. 4d. These phenomena mainly originate from the step dipole described as the Smoluchowski effect^41^,^42^,^43^. The step dipole, with an opposite direction to that of the surface dipole, induces a strong localized electric field at the step edge, resulting in Stark shifts of the adsorbed molecules near the step edge^41^. Since the SA structure of BTDA-TCNQ covers the step edge completely, the Stark shift in the SA structure is larger than that of the rearranged flat-lying adsorption structure. On the basis of a detailed analysis of the STS mapping data (see Supplementary Fig. 7), the Stark shifts of the unoccupied MO states of BTDA-TCNQ near the step edge are ∼−900 meV and ∼−300 meV for the SA structure and the rearranged flat-lying adsorption structure, respectively. These results strongly suggest that the localized electric fields induced by the step dipoles exert influences on the partial charge transfers from the step edges to the unoccupied MO states of BTDA-TCNQ. Considering the LUMO state of BTDA-TCNQ on the Au(111) terrace (at ∼+800 meV relative to the *E*_F_), the Stark shift of ∼−900 meV possibly induces the partial charge transfer to the LUMO state for the SA structure from the step edge, and the LUMO+1 state consequentially represents the nearest unoccupied MO state with respect to the *E*_F_ at the step edge (see Supplementary Fig. 7). The computationally estimated charge density difference map also strongly supports the partial charge transfer from the step edge to the molecule (see Supplementary Fig. 5). In addition, the STS spectrum and mapping images show that the unoccupied MO states for the SA structure of BTDA-TCNQ are highly hybridized with the Au surface at the step edge above ∼+1,800 meV with respect to the *E*_F_, forming quasi-1D electron dispersion along the step-edge line (see Fig. 4e).

### Electronic structures between the SMC and the Au surface

**Figure 5:**
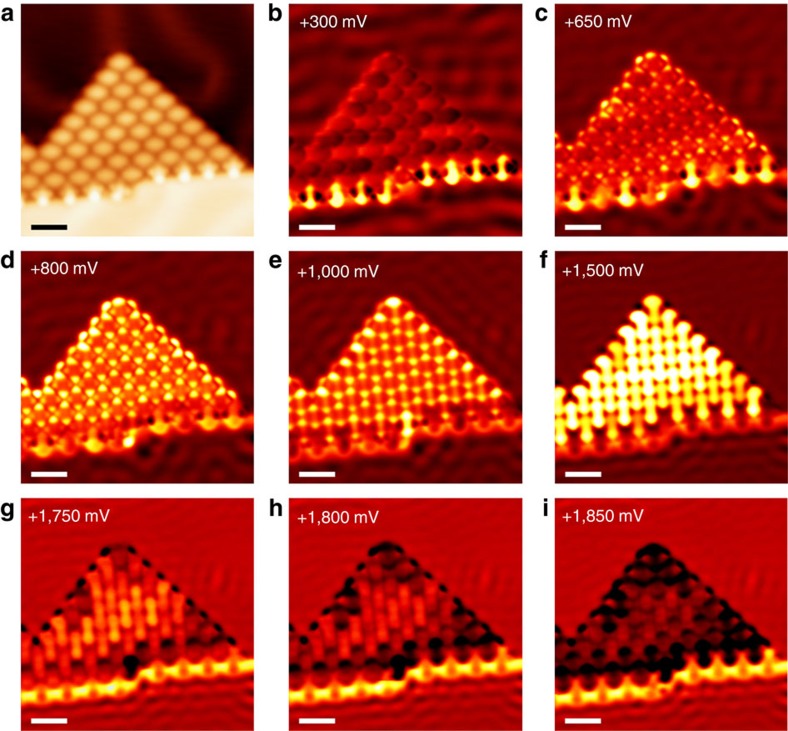
STS mapping of an SMC of BTDA-TCNQ on the Au(111) surface. (**a**) STM image of an SMC of BTDA-TCNQ on the Au(111) surface (*V*_s_=500 mV, *I*_t_=1.0 nA, *S*=2.0 nm). (**b**–**i**) STS mapping images of repeating measurements over the same surface area as that in **a**, with varying sample bias (*I*_t_=1.0 nA, *S*=2.0 nm). The Au(111) surface states are transparently observed through the SMC of BTDA-TCNQ in **b** (*V*_s_=300 mV) and **c** (*V*_s_=650 mV). The electron probability distributions of the MO states of BTDA-TCNQ are the most prominent features in **d** (*V*_s_=800 mV), **e** (*V*_s_=1,000 mV) and **f** (*V*_s_=1,500 mV). The SMC of BTDA-TCNQ exhibits a highly delocalized electron distribution which is confined within the boundaries of the SMC in **g** (*V*_s_=1,750 mV), **h** (*V*_s_=1,800 mV) and **i** (*V*_s_=1,850 mV). The quasi-1D electron dispersion along the step edge resulting from the hybridization of BTDA-TCNQ and Au at the step is also evident in **g**–**i**.

**Figure 6:**
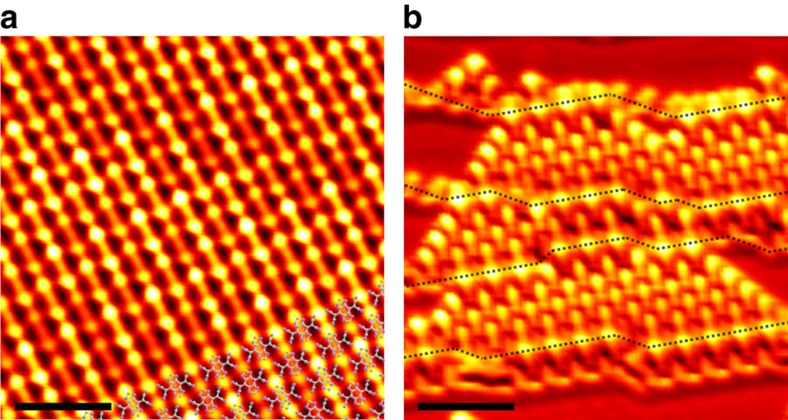
Widely uniform anisotropic MO states of BTDA-TCNQ/Au(111). (**a**,**b**) STS mapping images of an SMC of BTDA-TCNQ on the Au(111) surface in the energy range corresponding to the LUMO+1 state (*V*_s_=1,500 mV, *I*_t_=1.0 nA). Widely uniform anisotropic MO states of the SMC are clearly observed in **a** (*S*=3.0 nm), which can be extended over multiple surface steps and terraces as in **b** (*S*=5.0 nm). Step-edge lines are indicated with black dotted lines in **b**, revealing small sizes (∼5 nm in width) of the (111) facets.

The interfacial electronic structures between the SMC of BTDA-TCNQ and the Au(111) surface were also investigated as a function of energy and of local position, by repeating STS mapping over the same area of surface with the sample bias voltage varied between +300 mV and +2,000 mV (see Fig. 5). The interfacial electronic structures of the SMC on the Au(111) terrace are characterized into three main categories according to energy ranges. First, since the MO states of BTDA-TCNQ do not appear near the *E*_F_ as indicated in the STS spectrum (see Fig. 4a), the Au(111) surface states are transparently observed through the SMC in the lower energy region near +300 meV with respect to the *E*_F_ (see Fig. 5b,c). In this energy range, the STS mapping images exhibit strong standing-wave patterns of the surface-state electrons, which are continuous between two media (that is, the SMC and the Au(111) terrace) without scattering at the boundaries of the SMC^47^. Second, in the energy range corresponding to the MO states of BTDA-TCNQ, the electronic structures of the SMC can be attributed to the electron probability distributions of the MO states (see Fig. 5d–f). Thus, the STS mapping data reveal the localized MO states of the SMC independently of the Au(111) surface states, which also substantiates the weak adsorption nature of BTDA-TCNQ/Au(111) (ref. 48). Strongly scattered standing-wave patterns, which are caused by the reflection of the surface-state electrons at the boundaries of the SMC, are observed on the Au(111) terrace in this energy region. Compared with the lower energy region (near +300 meV), the observation of the scattering of surface-state electrons indicates that it is the localized MO states of the SMC that serve as potential walls for reflecting the surface-state electrons rather than the geometric footprint of the molecule^49^,^50^. Finally, above the highly scattering region of the surface-state electrons, the contribution of the MO states of BTDA-TCNQ decreases, as indicated in the STS spectrum (see Fig. 4a). In this higher energy region (near +1,900 meV with respect to the *E*_F_), the SMC exhibits a highly delocalized electron distribution, which is confined within the boundaries of the SMC (see Fig. 5g–i). The delocalized electron distribution shows a pattern of two-dimensional dispersive electronic states, and faintly scattered standing-wave patterns appear on the Au(111) terrace near the boundaries of the SMC. Considering the weak adsorption character of BTDA-TCNQ on the Au(111) terraces and the dissipation of the MO states in this higher energy region, it is the electronic states of Au(111) underneath the SMC that essentially contribute to the dispersive electronic states observed in the SMC^16^,^17^. On the other hand, the quasi-1D electron dispersion along the step edge resulting from the hybridization of BTDA-TCNQ and Au at the step is observed in this higher energy region separately from the terrace (see Fig. 5g–i), which implies that the surface steps modulate the dimensionality of the electron dispersion. Interestingly, these different types and dimensionalities of the interfacial electronic structures co-exist in the SMC of BTDA-TCNQ on the Au(111) surface. These results suggest that various types of electronic structures projected onto the SMC can be selectively available corresponding to the energy region and the local position of the system. Note that the SMC of BTDA-TCNQ on the Au(111) surface shows widely uniform anisotropic MO states in the energy region corresponding to the LUMO+1 state (see Fig. 6). Such anisotropic characteristics originate predominantly from the distribution of the LUMO+1 states along the S atoms and the uniaxial orientation of BTDA-TCNQ via the S–N intermolecular interactions, which are also maintained over multiple surface steps and terraces as shown in Fig. 6b. These results also imply that careful design of favourable intermolecular interactions facilitates the tailoring of direction and alignment of localized MO states in OTFs without the loss of structural integrity.

## Discussion

We present a well-designed system, BTDA-TCNQ on the Au surface, to demonstrate the formation of SMC with widely uniform interfacial structure and high adaptability on the metal surface. The strong non-bonding intermolecular interactions induced by the partial positive charge of the S atoms and the partial negative charge of the N atoms of BTDA-TCNQ, and the surface–molecule interactions on both surface steps and terraces enable the step-flow growth mode for the self-organized SMC domain. Owing to the well-balanced interfacial interactions, the SMC of BTDA-TCNQ on the Au surface can extend over multiple surface steps and terraces without losing its structural integrity, accompanying the unidirectional alignment of the MO states. This results in a highly uniform interfacial structure, even on the pre-annealed amorphous Au surface prepared on the glass substrate. In consideration of the typical amorphous Au electrodes widely used in electronics, our findings on the pre-annealed amorphous Au surface may have an impact on the field of organic electronics in terms of molecular orientation, alignment and ordering on the electrodes, which have profound effects on the electrical and optical properties^51^,^52^. In addition, selective availability of co-existing interfacial electronic structures projected onto the SMC of BTDA-TCNQ is one of the remarkable features of the SMC on the Au surface. We anticipate that the findings of this work will provide not only a deeper insight into the formation of OTFs with high film integrity at the O/M interfaces, but also a new way to tailor the properties of interfacial geometric and electronic structures for future applications of organic devices.

## Methods

### Low-temperature STM/STS experiments

All the experiments were performed using a low-temperature STM (Omicron GmbH) with an electrochemically etched tungsten tip in an ultrahigh-vacuum (UHV) chamber in which the base pressure was maintained at below 8.0 × 10^−11^ Torr. The Au(111) and amorphous Au surfaces were cleaned by several cycles of sputtering with argon ions (Ar^+^) and annealing at 800 K. Low-temperature STM surface scanning at atomic resolution was used to confirm the cleanliness of each Au surface. BTDA-TCNQ was synthesized and purified in accordance with a previous report^26^,^30^. Using a Knudsen cell, BTDA-TCNQ (in powdered form at room temperature) was thermally evaporated onto each Au surface under UHV conditions. Evaporation temperature was 473 K for BTDA-TCNQ, which was monitored using a K-type (alumel–chromel) thermocouple. The Au surface was maintained at room temperature during and after each thermal evaporation to confirm a thermodynamically stable phase (∼1 h), then cooled to 4.7 K. Note that the domain size of the SMC in the step-flow regime is mainly determined by the amount of adsorbed molecules at sub-monolayer coverage, which can be simply controlled by the deposition rate and time. In the deposition and annealing processes, no intentional temperature control was performed for the Au surface, and the substrate was merely located in the room temperature chamber. When the sample was immediately cooled to 4.7 K (cooling time of ∼1 h) even without annealing time after deposition, there were no significant changes observed in the formation of SMC as well following the step-flow growth scheme. The cooled sample was also not damaged if the sample temperature repeatedly increased up to room temperature in the UHV chamber.

All STM and STS measurements were performed at 4.7 K, and STS signals were measured with lock-in detection by applying a modulation of 50 mV (r.m.s.) to the sample bias voltage at 797 Hz. Note that all STS spectra were obtained at a fixed tip-sample separation (feed-back off), whereas STS mapping was performed in the constant current mode, with the sample bias voltage varied from +300 mV to +2,000 mV.

### DFT calculations

We used vdW-TS method^53^ implemented in the Vienna *Ab initio* Simulation Package (VASP) code^54^,^55^, which can take account of van der Waals interactions, to examine the formation of SMC. The core electrons were replaced by projector-augmented wave pseudopotentials^56^, expanded in a basis set of plane waves up to a cutoff energy of 400 eV. The substrate-free DFT calculations were performed, based on the experimental observations of weak surface–molecule interactions. The minimum-size supercell including one molecule was used according to the experimentally observed periodicity of the two-dimensional intermolecular network. For comparisons among a variety of initial geometries, various molecular orientations rotated by 10° increments were prepared with respect to the experimentally proposed molecular arrangement. The calculations using DFT-D2 method^57^ were also performed to verify the computational reliability, leading to a good agreement with the results obtained using vdW-TS method. During ionic relaxation, the molecular geometry in a planar fashion was maintained by symmetry constraints, but the volume and shape of the slab were freely adjusted toward a local potential minimum. To ensure two-dimensional molecular architecture, we initially separated the periodically replicated slabs with a large vacuum region of 15 Å. The resultant vacuum regions of the optimized slabs were about 6.0 Å, in which the interactions between the molecular layers are expected to be negligible in obtaining proper geometries and energies of the intermolecular network structures of BTDA-TCNQ. Ionic (electronic) relaxations were performed until atomic forces (energies) were less than 0.05 eV Å^−1^ (10^−7^ eV). The k-point sampling of the Brillouin zone was performed with 8 × 8 × 1 Γ-centred grids in the periodic DFT calculations.

As regards the adsorption structure of BTDA-TCNQ at the step edge of Au(111), we used a (6 × 1)-Au(455) supercell to investigate the adsorption of isolated BTDA-TCNQ molecule, which provides the nearest interatomic distance of longer than 8 Å along the step-edge line and the large distance from the molecule to the next step edge longer than 15 Å. The slab model was composed of six Au layers and vacuum region of ∼20 Å, in which the two bottom layers were fixed in their bulk positions. Bader population analysis^58^ was also performed to evaluate the partial charge transfer from the surface to the molecule at the step edge. Dipole correction was applied to avoid undesired interactions between periodic slab images. The k-point sampling of the Brillouin zone was performed with 2 × 2 × 1 Γ-centred grids in the periodic DFT calculations.

### FET fabrication and characterization

BTDA-TCNQ was incorporated into the FET configuration with a bottom-gate and bottom-contact structure. A highly doped n-type Si wafer with a thermally grown SiO_2_ dielectric layer (of ∼300 nm thickness) was used as a substrate. The Au source and drain electrodes (of ∼40 nm thickness) were deposited onto the pre-cleaned substrate by thermal evaporation in a vacuum chamber, and then annealed at 300 °C for ∼1 h in a N_2_-filled glove-box. The channel length and width between the Au source and drain electrodes were 50 μm and 975 μm, respectively. After cooling the substrate to room temperature, BTDA-TCNQ (of ∼50 nm thickness) was evaporated at the rate of 0.1 Å s^−1^. The transfer and output characteristics (see Supplementary Fig. 6) were measured using a semiconductor parameter analyser (Agilent B1500A) in the N_2_-filled glove-box at room temperature.

## Additional information

**How to cite this article**: Kim, J.-H. *et al.* Seamless growth of a supramolecular carpet. *Nat. Commun.* 7:10653 doi: 10.1038/ncomms10653 (2016).

## Supplementary Material

Supplementary Information Supplementary Figures 1-7 and Supplementary References

## Figures and Tables

**Table 1:** Relative energies, adsorption energies and optimized nearest atomic distances of Ṡ̇̇Au and –CṄ̇̇Au of four adsorption models, ‘SA1', ‘SA2', ‘SA3' and ‘SA4'.

	**SA1**	**SA2**	**SA3**	**SA4**
*E*_rel_ (eV)	0.00	0.17	0.52	0.53
*E*_ads_ (eV)	2.64	2.47	2.12	2.11
				
*Step edge*				
Ṡ̇̇Au (Å)	3.12 (× 2)	2.54		
–CṄ̇̇Au (Å)	2.18	2.56	2.31	2.34
				
*Terrace*				
Ṡ̇̇Au (Å)	3.34	3.33	3.61	3.69
–CṄ̇̇Au (Å)	3.17	2.36	2.68	3.14

The optimized distances at the step edge and on the terrace are presented, respectively. In ‘SA1', a bidentate fashion of Ṡ̇̇Au at the step edge is denoted with ‘ × 2'.

## References

[b1] Park S. H. *et al.* Bulk heterojunction solar cells with internal quantum efficiency approaching 100%. Nat. Photon. 3, 297–302 (2009).

[b2] Sekitani T. *et al.* Stretchable active matrix organic light-emitting diode display using printable elastic conductors. Nat. Mater. 8, 494–499 (2009). 19430465 10.1038/nmat2459

[b3] Sekitani T., Zschieschang U., Klauk H. & Someya T. Flexible organic transistors and circuits with extreme bending stability. Nat. Mater. 9, 1015–1022 (2010). 21057499 10.1038/nmat2896

[b4] Zhang W. Supramolecular linear heterojunction composed of graphite-like semiconducting nanotubular segments. Science 334, 340–343 (2011). 22021852 10.1126/science.1210369

[b5] Uoyama H., Goushi K., Shizu K., Nomura H. & Adachi C. Highly efficient organic light-emitting diodes from delayed fluorescence. Nature 492, 234–238 (2012). 23235877 10.1038/nature11687

[b6] Kim R. H. *et al.* Non-volatile organic memory with sub-millimetre bending radius. Nat. Commun. 5, 3583 (2014). 24709956 10.1038/ncomms4583

[b7] Forrest S. R. Ultrathin organic films grown by organic molecular beam deposition and related techniques. Chem. Rev. 97, 1793–1896 (1997). 11848893 10.1021/cr941014o

[b8] Ishii H., Sugiyama K., Ito E. & Seki K. Energy level alignment and interfacial electronic structures at organic/metal and organic/organic interfaces. Adv. Mater. 11, 605–625 (1999).

[b9] Hooks D. E., Fritz T. & Ward M. D. Epitaxy and molecular organization on solid substrates. Adv. Mater. 13, 227–241 (2001).

[b10] Zhu X.-Y. Electronic structure and electron dynamics at molecule-metal interfaces: implications for molecule-based electronics. Surf. Sci. Rep. 56, 1–83 (2004).

[b11] Braun S., Salaneck W. R. & Fahlman M. Energy-level alignment at organic/metal and organic/organic interfaces. Adv. Mater. 21, 1450–1472 (2009).

[b12] Kawano K. & Adachi C. Evaluating carrier accumulation in degraded bulk heterojunction organic solar cells by a thermally stimulated current technique. Adv. Funct. Mater. 19, 3934–3940 (2009).

[b13] Yamane H., Kanai K., Ouchi Y., Ueno N. & Seki K. Impact of interface geometric structure on organic–metal interface energetics and subsequent films electronic structure. J. Electron Spectrosc. Relat. Phenom. 174, 28–34 (2009).

[b14] Ma H., Yip H.-L., Huang F. & Jen A. K.-Y. Interface engineering for organic electronics. Adv. Funct. Mater. 20, 1371–1388 (2010).

[b15] Barth J. V., Costantini G. & Kern K. Engineering atomic and molecular nanostructures at surfaces. Nature 437, 671–679 (2005). 16193042 10.1038/nature04166

[b16] Temirov R., Soubatch S., Luican A. & Tautz F. S. Free-electron-like dispersion in an organic monolayer film on a metal substrate. Nature 444, 350–353 (2006). 17108961 10.1038/nature05270

[b17] Gonzalez-Lakunza N. *et al.* Formation of dispersive hybrid bands at an organic-metal interface. Phys. Rev. Lett. 100, 156805 (2008). 18518142 10.1103/PhysRevLett.100.156805

[b18] Bartels L. Tailoring molecular layers at metal surfaces. Nat. Chem. 2, 87–95 (2010). 21124397 10.1038/nchem.517

[b19] Tseng T.-C. *et al.* Charge-transfer-induced structural rearrangements at both sides of organic/metal interfaces. Nat. Chem. 2, 374–379 (2010). 20414237 10.1038/nchem.591

[b20] Grill L. *et al.* Nano-architectures by covalent assembly of molecular building blocks. Nat. Nanotechnol. 2, 687–691 (2007). 18654406 10.1038/nnano.2007.346

[b21] Zwaneveld N. A. A. *et al.* Organized formation of 2D extended covalent organic frameworks at surfaces. J. Am. Chem. Soc. 130, 6678–6679 (2008). 18444643 10.1021/ja800906f

[b22] Abdurakhmanova N. *et al.* Stereoselectivity and electrostatics in charge-transfer Mn- and Cs-TCNQ_4_ networks on Ag(100). Nat. Commun. 3, 940 (2012). 22760639 10.1038/ncomms1942

[b23] Kley C. S. *et al.* Highly adaptable two-dimensional metal–organic coordination networks on metal surfaces. J. Am. Chem. Soc. 134, 6072–6075 (2012). 22458838 10.1021/ja211749b

[b24] Li Y. *et al.* Coordination and metalation bifunctionality of Cu with 5, 10, 15, 20-tetra (4-pyridyl) porphyrin: toward a mixed-valence two-dimensional coordination network. J. Am. Chem. Soc. 134, 6401–6408 (2012). 22414052 10.1021/ja300593w

[b25] Floris A., Comisso A. & Vita A. D. Fine-tuning the electrostatic properties of an alkali-linked organic adlayer on a metal substrate. ACS Nano 7, 8059–8065 (2013). 23968290 10.1021/nn403274s

[b26] Schnadt J. *et al.* Extended one-dimensional supramolecular assembly on a stepped surface. Phys. Rev. Lett. 100, 046103 (2008). 18352306 10.1103/PhysRevLett.100.046103

[b27] Schnadt J. *et al.* Interplay of adsorbate-adsorbate and adsorbate-substrate interactions in self-assembled molecular surface nanostructures. Nano Res. 3, 459–471 (2010).

[b28] Li B. *et al.* Self-assembled air-stable supramolecular porous networks on graphene. ACS Nano 7, 10764–10772 (2013). 24206021 10.1021/nn4039047

[b29] Yamashita Y., Suzuku T., Mukai T. & Saito G. Preparation and properties of a tetracyanoquinodimethane fused with 1,2,5-thiadiazole units. J. Chem. Soc. Chem. Commun. 1985, 1044–1045 (1985).

[b30] Torrance J. B. The difference between metallic and insulating salts of tetracyanoquinodimethone (TCNQ): how to design an organic metal. Acc. Chem. Res. 12, 79–86 (1979).

[b31] Alves H., Molinari A. S., Xie H. & Morpurgo A. F. Metallic conduction at organic charge-transfer interfaces. Nat. Mater. 7, 574–580 (2008). 18552852 10.1038/nmat2205

[b32] Kirtley J. R. & Mannhart J. Organic electronics: when TTF met TCNQ. Nat. Mater. 7, 520–521 (2008). 18552848 10.1038/nmat2211

[b33] Suzuki T. *et al.* Clathrate formation and molecular recognition by novel chalcogen-cyano interactions in tetracyanoquinodimethanes fused with thiadiazole and selenadiazole rings. J. Am. Chem. Soc. 114, 3034–3043 (1992).

[b34] Suzuki T., Fukushima T., Yamashita Y. & Miyashi T. An absolute asymmetric synthesis of the [2+2] cycloadduct via single crystal-to-single crystal transformation by charge-transfer excitation of solid-state molecular complexes composed of arylolefins and bis[1,2,5]thiadiazolotetracyanoquinodimethane. J. Am. Chem. Soc. 116, 2793–2803 (1994).

[b35] Vlieg E. Understanding crystal growth in vacuum and beyond. Surf. Sci. 500, 458–474 (2002).

[b36] Wagner S. R., Lunt R. R. & Zhang P. Anisotropic crystalline organic step-flow growth on deactivated Si surfaces. Phys. Rev. Lett. 110, 086107 (2013). 23473173 10.1103/PhysRevLett.110.086107

[b37] Aragó J., Sancho-García J. C., Ortí E. & Beljonne D. *Ab initio* modeling of donor–acceptor interactions and charge-transfer excitations in molecular complexes: the case of terthiophene–tetracyanoquinodimethane. J. Chem. Theory Comput. 7, 2068–2077 (2011). 26606478 10.1021/ct200203k

[b38] Sini G., Sears J. S. & Brédas J.-L. Evaluating the performance of DFT functionals in assessing the interaction energy and ground-state charge transfer of donor/acceptor complexes: tetrathiafulvalene-tetracyanoquinodimethane (TTF-TCNQ) as a model case. J. Chem. Theory Comput. 7, 602–609 (2011). 26596294 10.1021/ct1005517

[b39] Beesley D. J. *et al.* Sub-15-nm patterning of asymmetric metal electrodes and devices by adhesion lithography. Nat. Commun. 5, 3933 (2014). 24861953 10.1038/ncomms4933 PMC4050269

[b40] Cho H. *et al.* Replication of flexible polymer membranes with geometry-controllable nano-apertures via a hierarchical mould-based dewetting. Nat. Commun. 5, 3137 (2014). 24451920 10.1038/ncomms4137

[b41] Wandelt K. Properties and influence of surface defects. Surf. Sci. 251, 387–395 (1991).

[b42] Avouris P., Lyo I.-W. & Molinàs-Mata P. STM studies of the interaction of surface state electrons on metals with steps and adsorbates. Chem. Phys. Lett. 240, 423–428 (1995).

[b43] Kamna M. M., Stranick S. J. & Weiss P. S. Imaging substrate-mediated interactions. Isr. J. Chem. 36, 59–62 (1996).

[b44] Chen W., Madhavan V., Jamneala T. & Crommie M. F. Scanning tunneling microscopy observation of an electronic superlattice at the surface of clean gold. Phys. Rev. Lett. 80, 1469–1472 (1998).

[b45] Kim J.-H. *et al.* Direct observation of adsorption geometry for the van der Waals adsorption of a single π-conjugated hydrocarbon molecule on Au (111). J. Chem. Phys. 140, 074709 (2014). 24559362 10.1063/1.4864458

[b46] Wisendanger R. Scanning Probe Microscopy and Spectroscopy: Methods and Applications Cambridge Univ. Press (1994).

[b47] Repp J., Meyer G. & Rieder K.-H. Snell's law for surface electrons: refraction of an electron gas imaged in real space. Phys. Rev. Lett. 92, 036803 (2004). 14753892 10.1103/PhysRevLett.92.036803

[b48] Soe W.-H., Manzano C., De Sarkar A., Chandrasekhar N. & Joachim C. Direct observation of molecular orbitals of pentacene physisorbed on Au (111) by scanning tunneling microscope. Phys. Rev. Lett. 102, 176102 (2009). 19518800 10.1103/PhysRevLett.102.176102

[b49] Gross L. *et al.* Scattering of surface state electrons at large organic molecules. Phys. Rev. Lett. 93, 056103 (2004). 15323715 10.1103/PhysRevLett.93.056103

[b50] Seufert K. *et al.* Controlled interaction of surface quantum-well electronic states. Nano Lett. 13, 6130–6135 (2013). 24245663 10.1021/nl403459m

[b51] Dimitrakopoulos C. D. & Malenfant P. R. L. Organic thin film transistors for large area electronics. Adv. Mater. 14, 99–117 (2002).

[b52] Yokoyama D. Molecular orientation in small-molecule organic light-emitting diodes. J. Mater. Chem. 21, 19187–19202 (2011).

[b53] Tkatchenko A. & Scheffler M. Accurate molecular van der Waals interactions from ground-state electron density and free-atom reference data. Phys. Rev. Lett. 102, 073005 (2009). 19257665 10.1103/PhysRevLett.102.073005

[b54] Kresse G. & Hafner J. *Ab initio* molecular dynamics for liquid metals. Phys. Rev. B 47, 558–561 (1993). 10.1103/physrevb.47.558 10004490

[b55] Kresse G. & Furthmüller J. Efficient iterative schemes for *ab initio* total-energy calculations using a plane-wave basis set. Phys. Rev. B 54, 11169–11186 (1996). 10.1103/physrevb.54.11169 9984901

[b56] Kresse G. & Joubert D. From ultrasoft pseudopotentials to the projector augmented-wave method. Phys. Rev. B 59, 1758–1775 (1999).

[b57] Grimme S. Semiempirical GGA-type density functional constructed with a long-range dispersion correction. J. Comp. Chem. 27, 1787–1799 (2006). 16955487 10.1002/jcc.20495

[b58] Tang W., Sanville E. & Henkelman G. A grid-based Bader analysis algorithm without lattice bias. J. Phys. Condens. Matter 21, 084204 (2009). 21817356 10.1088/0953-8984/21/8/084204

