# Supplementary material for: Seamless growth of a supramolecular carpet
Source: Nat Commun. 2016 Feb 3;7:10653. doi: 10.1038/ncomms10653 (PMC4742838; doi:10.1038/ncomms10653)
Supplement: Supplementary Information — Supplementary Figures 1-7 and Supplementary References [file ncomms10653-s1.pdf]

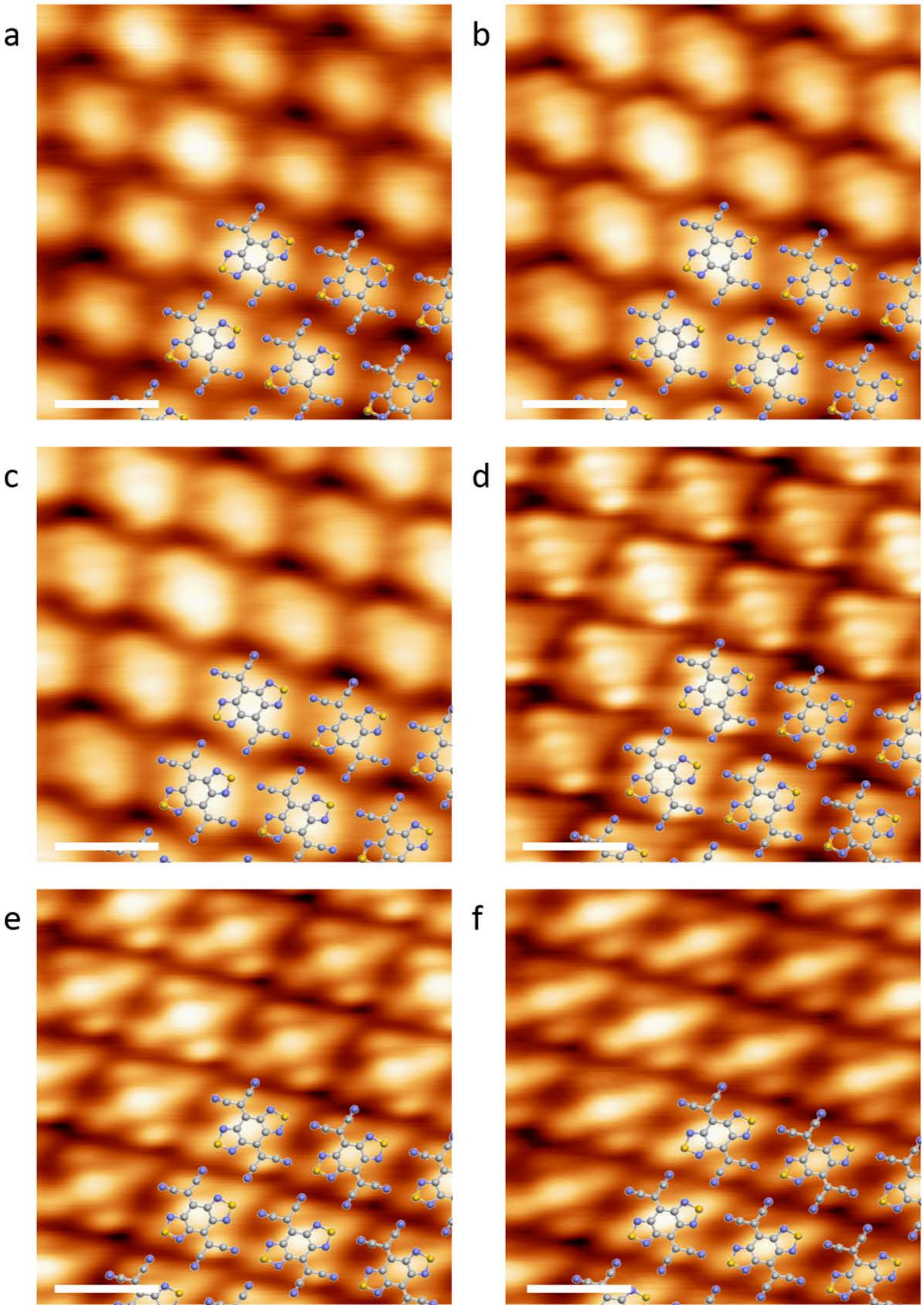

Supplementary Figure 1. Bias dependence in STM imaging of BTDA-TCNQ/Au(111). a-f, STM images of repeating measurements over the same surface area with varying sample bias (I_t = 1.0 nA, S = 1.0 nm). The STM images were obtained at V_s of -2000 mV (**a**), -500 mV (**b**), +500 mV (**c**), +1000 mV (**d**), +1500 mV (**e**), and +2000 mV (**f**). The distinct electronic structures of the unoccupied MO states lead to strong bias dependence in STM imaging with positive sample bias.

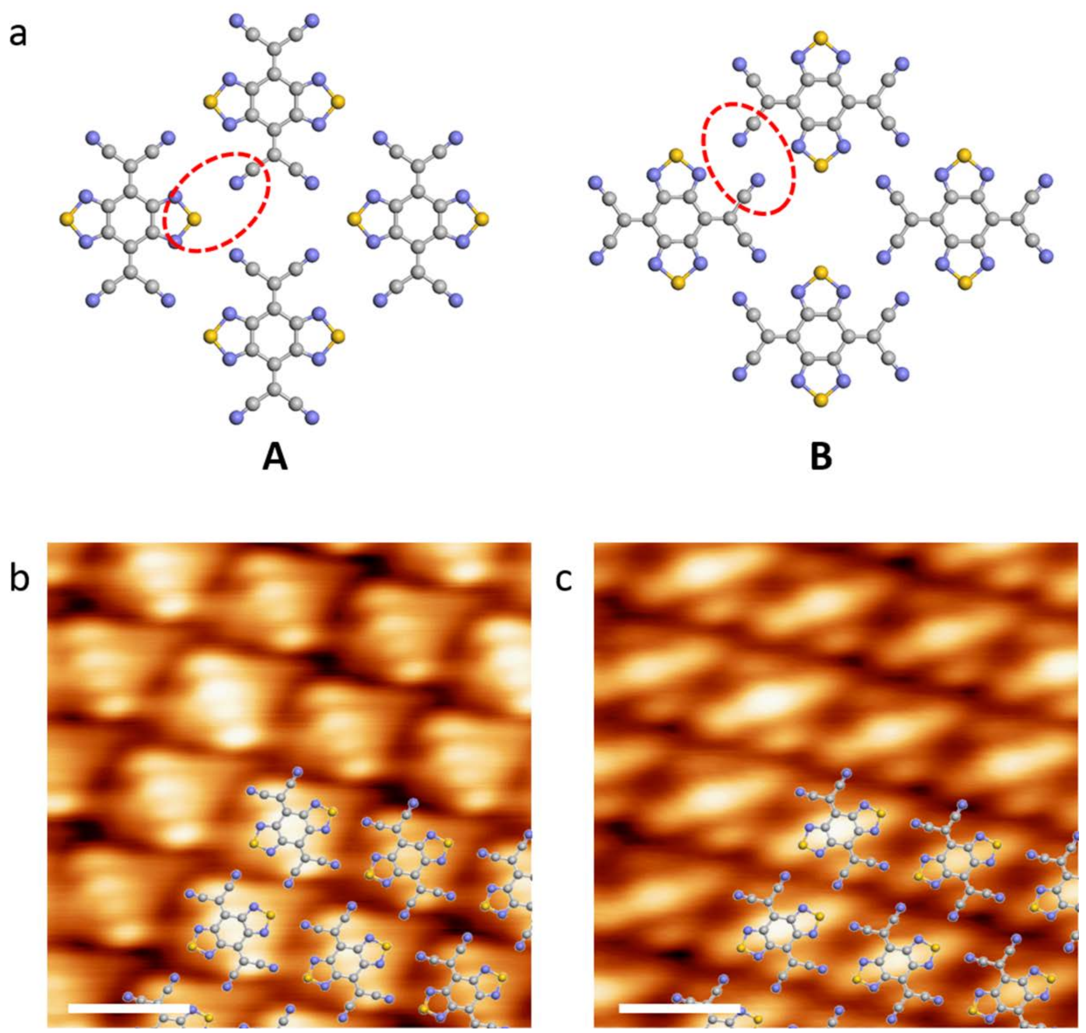

Supplementary Figure 2. Molecular orientation of BTDA-TCNQ in SMC formation. a, Two hypothetical models of the unit cell structure of the BTDA-TCNQ network (designated “A” and “B”). Since the lattice constants of the BTDA-TCNQ network are $a = b = 10.0 \pm 1.0$ Å and $\gamma = 80 \pm 5°$, two arrangements of the unit cell structure are hypothetically possible. Shortest intermolecular atomic distances are indicated by red dashed circles. The distance between the electronegative –CN groups in “B” (2.70 Å) is significantly shorter than in “A” (4.10 Å), resulting in larger repulsive interactions between the neighbouring molecules in “B”. In addition, the attractive S-N intermolecular interactions are enhanced in “A”, because the distance for the S-N contact of “A” (2.80 Å) is considerably shorter than of “B” (3.20 Å). We approximately estimated such intermolecular atomic separation by arranging computationally optimised isolated molecules in the experimentally observed lattice, for which the periodic DFT calculation was performed using a cubic cell with a side length of 25 Å. The unit cell structure of “A” also shows good consistency with the crystal structure of BTDA-TCNQ, in which the S-N contact distance is 3.04 Å[1]. **b,** STM image of the BTDA-TCNQ network (V_s = 1000 mV, I_t = 1.0 nA, S = 1.0 nm), showing three nodal planes in the TCNQ parts. **c,** STM image of the same surface area as **b** at different sample bias (V_s = 2000 mV, I_t = 1.0 nA, S = 1.0 nm). The overlays in **b,c** represent the proposed model “A”. Note that STM images show three nodal planes of TCNQ on the Au(111) surface at a sample bias of ~1000 mV[2,3], which strongly supports the unit cell structure of “A”.

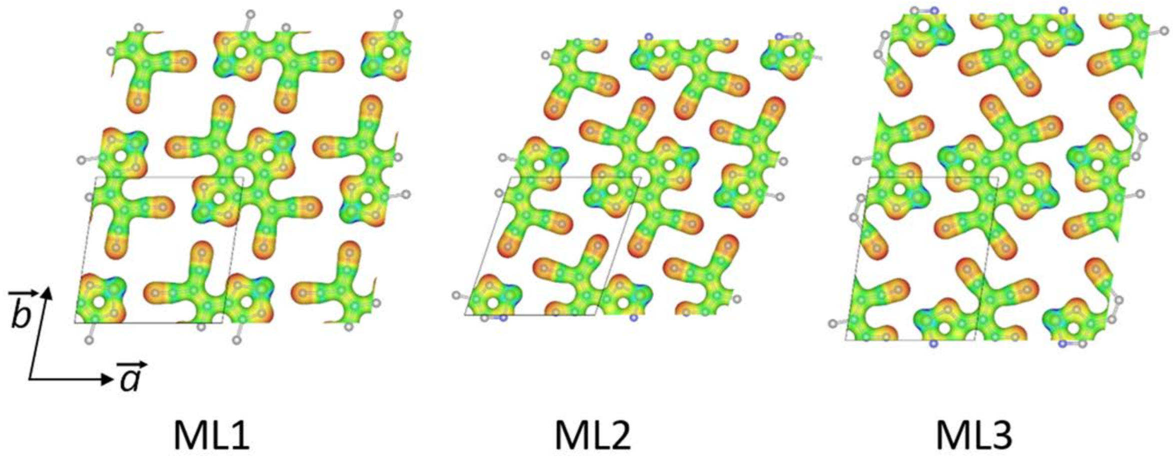

		ML1	ML2	ML3
vdW-TS	E_{rel} (eV)	0.00	0.34	0.58
	E_{bind} (eV/molecule)	0.71	0.36	0.12
	Lattice parameters (a, b, γ)	9.87 Å, 9.87 Å, 81.5°	9.20 Å, 10.27 Å, 71.4°	8.65 Å, 11.08 Å, 81.6°
DFT-D2	E_{rel} (eV)	0.00	0.32	0.54
	E_{bind} (eV/molecule)	0.68	0.36	0.14
	Lattice parameters (a, b, γ)	9.89 Å, 9.89 Å, 81.5°	9.17 Å, 10.23 Å, 71.3°	8.65 Å, 11.03 Å, 81.7°

Supplementary Figure 3. Electrostatic potential maps of BTDA-TCNQ molecular layers corresponding to various molecular orientations. The electrostatic potential maps for the optimised geometries (local potential minima) of BTDA-TCNQ molecular layers corresponding to various molecular orientations. Blue-to-red corresponds to positive-to-negative charges (iso-value = 0.01 e/bohr^3). Relative energies, binding energies, and optimised lattice parameters are presented in bottom table, which were calculated using two different functionals, vdW-TS and DFT-D2. The experimentally observed azimuthal orientation is same as the most stable "ML1" structure.

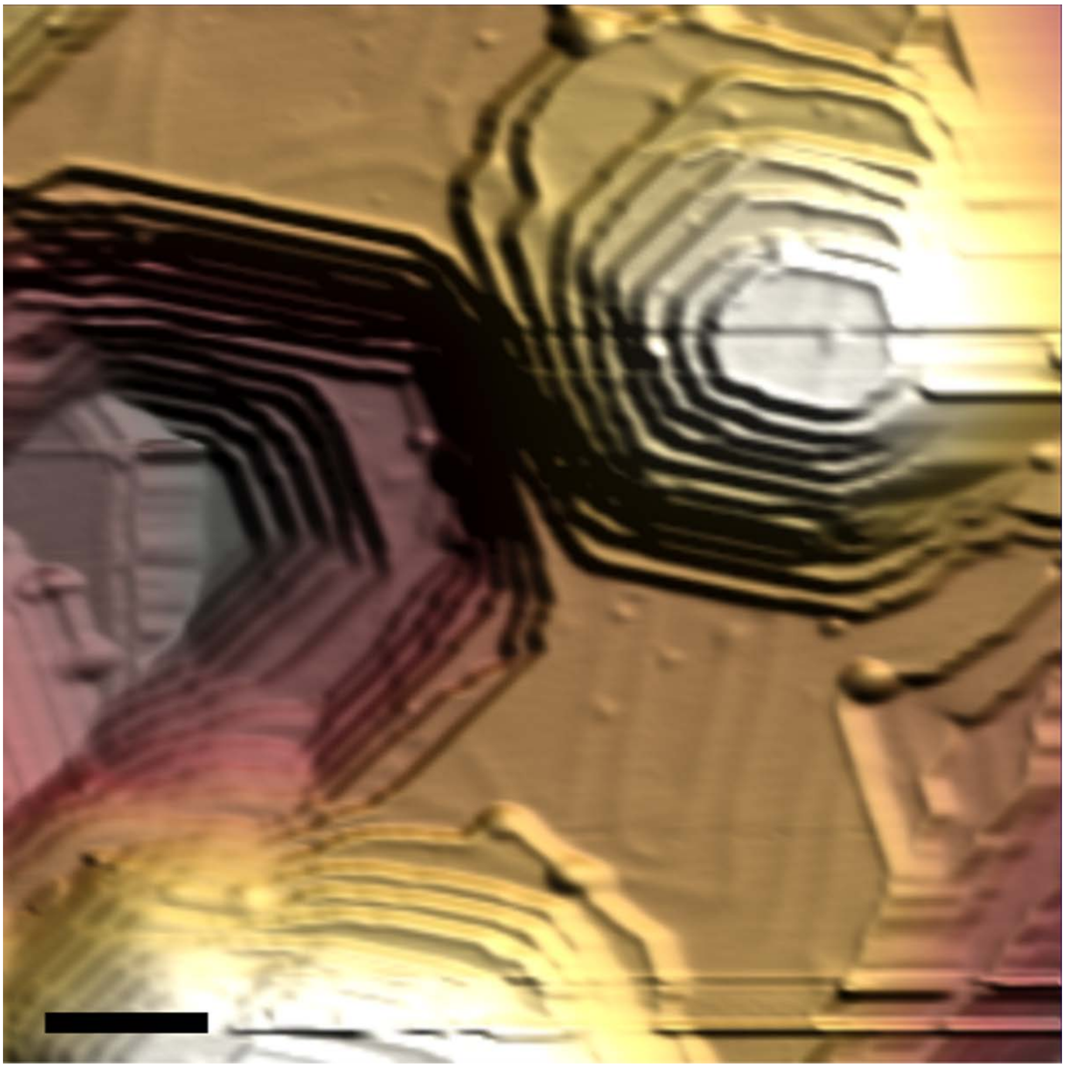

Supplementary Figure 4. Pre-annealed amorphous Au surface. STM image of a pre-annealed amorphous Au sample (V_s = +3000 mV, I_t = 0.5 nA, S = 10 nm), which shows the close-packed (111) facet with irregular steps and terraces. Since the energies of metal surfaces are naturally lowered when the surfaces are replaced by facets with lower-index orientations, implying that even the low temperature equilibrium shapes of metal crystals are heavily faceted[4]. Thus, not atomically vertical multilayer steps but rather small (111) facets at least appear between adjacent steps even on the pre-annealed amorphous Au surface.

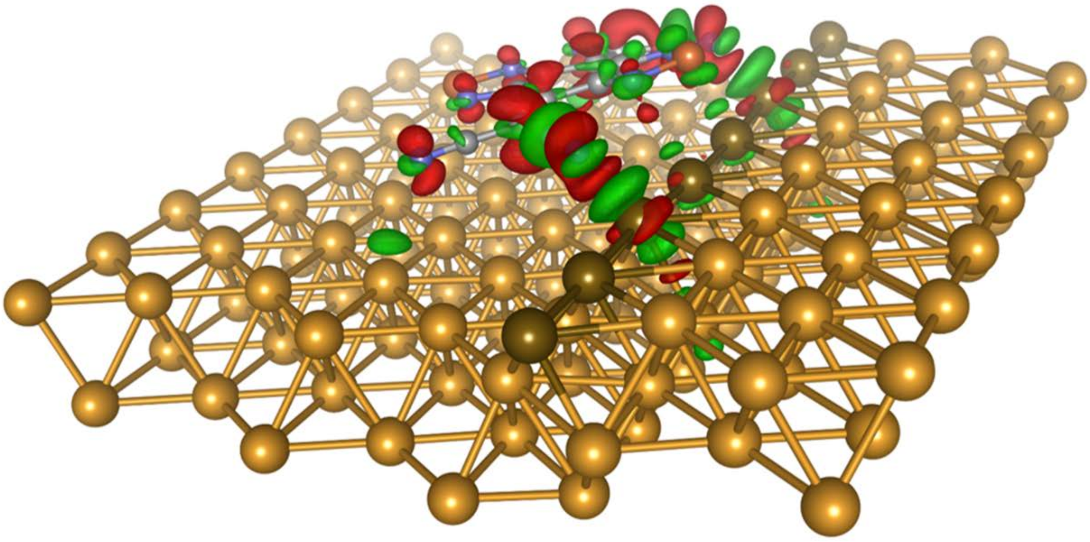

Supplementary Figure 5. Charge density difference map for “SA1”. The charge density difference map of the BTDA-TCNQ molecule adsorbed at the step edge of Au(111), “SA1”. Red and green regions correspond to electron-gain and -depletion regions, respectively (iso-value = ±0.002 e/bohr^3), which clearly indicate the partial charge transfer from the surface to the molecule. The corresponding amount of the partial charge transfer evaluated by Bader population analysis is 0.68*e*.

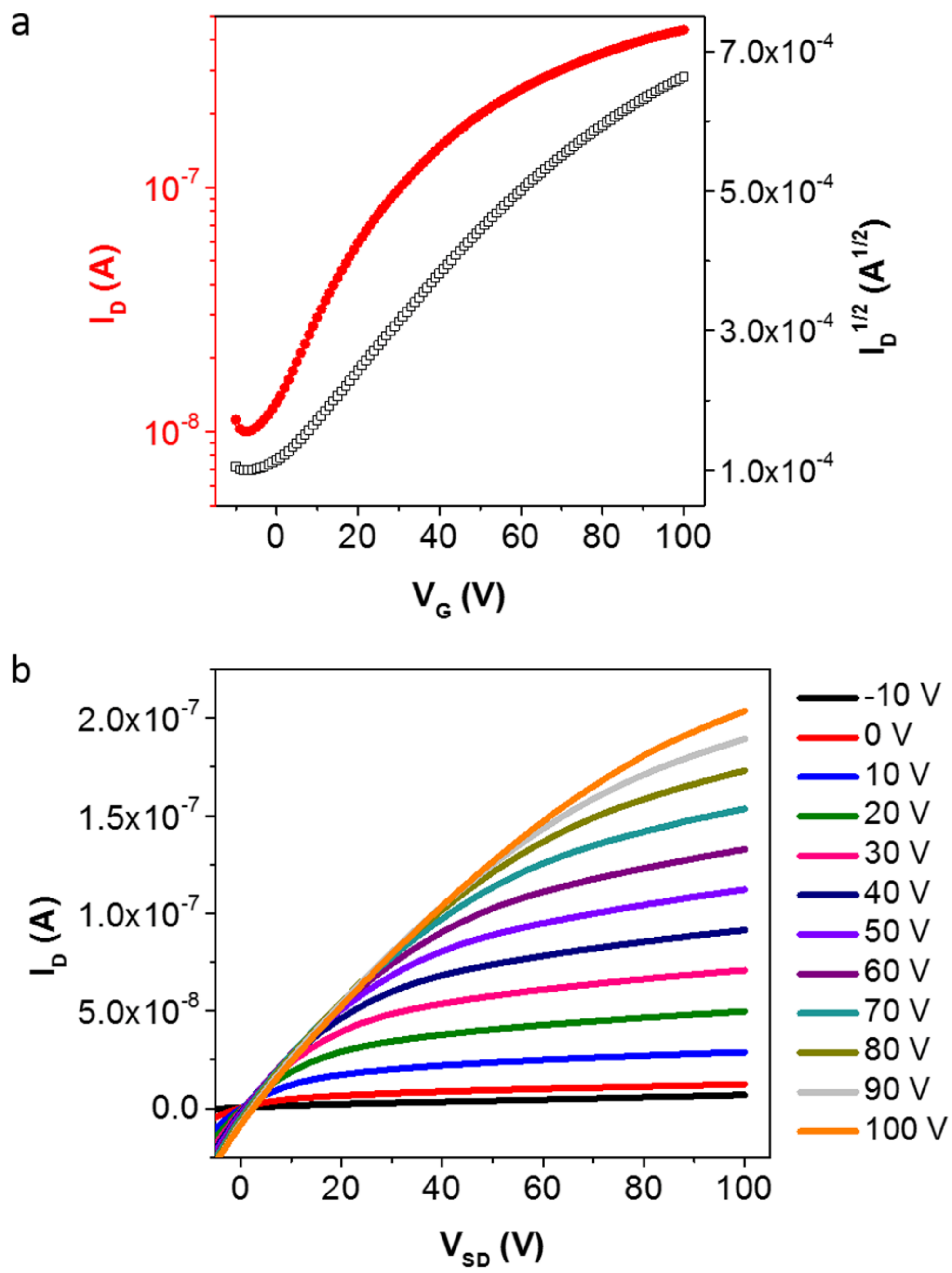

Supplementary Figure 6. FET characteristics. a, Transfer characteristics of BTDA-TCNQ in the FET configuration in log scale (indicated by filled red circles), and in square-root scale (indicated by hollow black squares). **b,** Output characteristics of BTDA-TCNQ in the FET configuration. An average electron mobility of ~10^{-3} $cm^2 \cdot V^{-1} \cdot s^{-1}$, and an on-to-off current ratio of ~50 were observed in the FET configuration. It is worth noting that the interfaces between n-type organic semiconductors and SiO_2 dielectrics induce trapping of electrons, which is associated with a loss of FET activity[5]. Thus, our results indicate that BTDA-TCNQ has a sufficiently high electron affinity to show n-type semiconducting behaviour, even on the SiO_2 dielectric layer. The electron mobility of this system is higher than that of the deposited TCNQ film and the TNCQ single crystal on SiO_2[6,7].

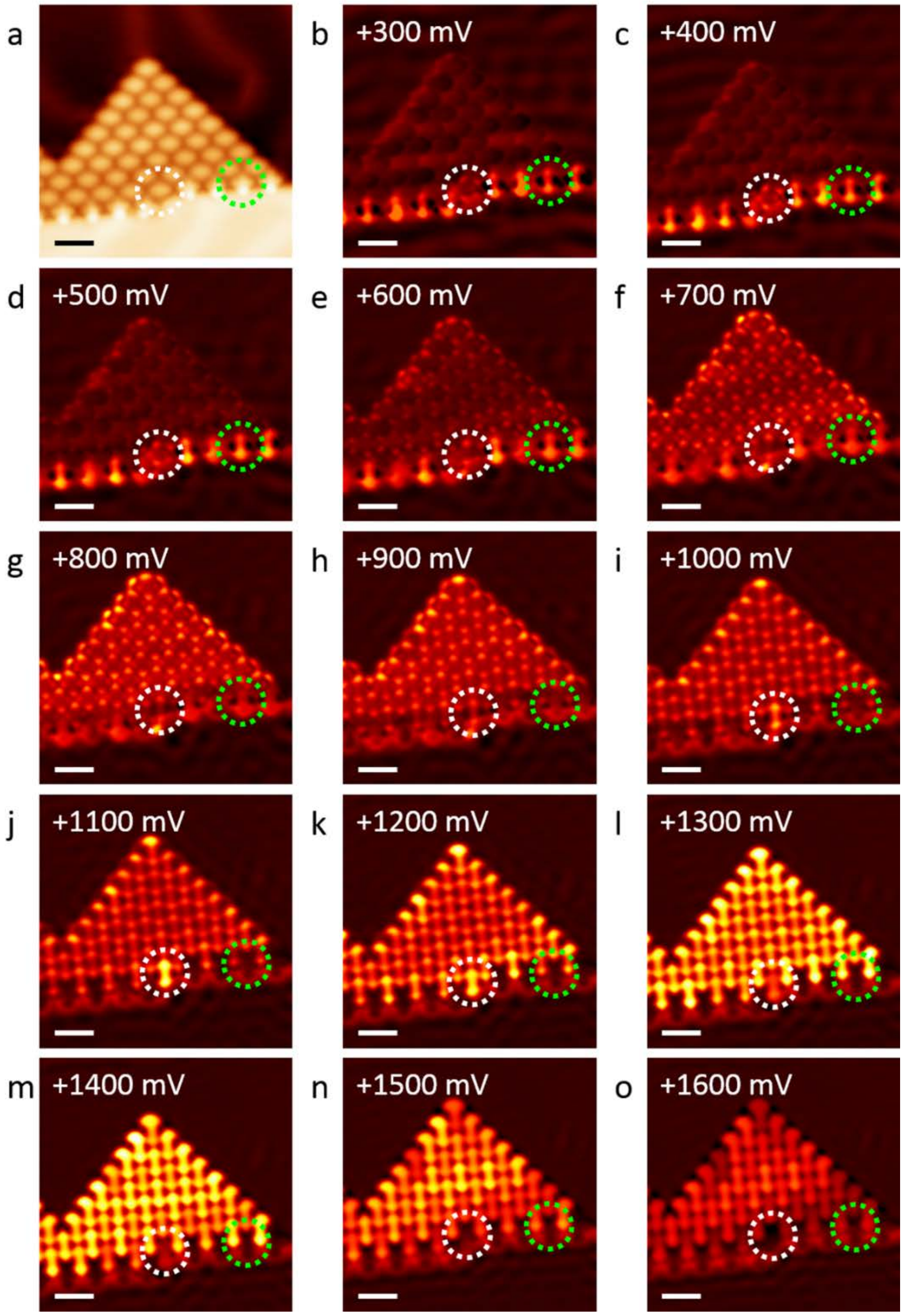

Supplementary Figure 7. Stark-shifts of the BTDA-TCNQ molecules near the Au(111) step edge. a, STM image of the SMC of BTDA-TCNQ on the Au(111) surface (V_s = 500 mV, I_t = 1.0 nA, S = 2.0 nm). **b-o,** STS mapping images of repeating measurements over the same surface area as that in **a**, with sample bias varying from +300 mV to +1600 mV (I_t = 1.0 nA, S = 2.0 nm). Note that the Z colour scales are identical in **b-o**. The BTDA-TCNQ molecules with the rearranged flat-lying adsorption structure (indicated by white dotted circle) and the slanting adsorption structure (indicated by green dotted circle) show Stark shifts near the Au(111) step edge. Considering electron probability distributions and scales, the Stark shifts of the unoccupied MO states of BTDA-TCNQ near the step edge are ~-300 meV and ~-900 meV for the rearranged flat-lying adsorption structure and the slanting adsorption structure, respectively.

Supplementary References

1 Yamashita, Y., Suzuki, T., Saito, G. & Muka, T. Highly conductive complexes of bis-1, 2, 5-thiadiazolo-tetracyanoquinodimethan (BTDA-TCNQ) with amines. *Chem. Lett.* **14**, 1759-1762 (1985).

2 Gonzalez-Lakunza, N. *et al.* Formation of dispersive hybrid bands at an organic-metal interface. *Phys. Rev. Lett.* **100**, 156805 (2008).

3 Tirrebte, I. F., Franke, K. J. & Pascual, J. I. Structure and electronic configuration of tetracyanoquinodimethane layers on a Au(111) surface. *Int. J. Mass Spectrosc.* **277**, 269-273 (2008).

4 Frenken, J. W. M. & Stoltze, P. Are vicinal metal surfaces stable? *Phys. Rev. Lett.* **82**, 3500-3503 (1999).

5. Chua, L.-L. *et al.* General observation of n-type field-effect behaviour in organic semiconductors. *Nature* **434**, 194-199 (2005).

6. Briseno, A. L. *et al.* Patterning organic single-crystal transistor arrays. *Nature* **444**, 913-917 (2006).

7. Mori, T. Molecular materials for organic field-effect transistors. *J. Phys.: Condens. Matter* **20**, 184010 (2008).
